# A New Approach to Electrical Fault Detection in Urban Structures Using Dynamic Programming and Optimized Support Vector Machines

**DOI:** 10.3390/s25072215

**Published:** 2025-04-01

**Authors:** Reynaldo Villarreal, Sindy Chamorro-Solano, Yolanda Vega-Sampayo, Carlos Alejandro Espejo, Steffen Cantillo, Luis Gaviria, Jheifer Paez, Carlos Ochoa, Silvia Moreno, Claudet Polo, Roberto Pestana-Nobles, Camilo Montoya

**Affiliations:** 1 Centro de Investigación, Desarrollo Tecnológico e Innovación en Inteligencia Artificial y Robótica, AudacIA, Universidad Simón Bolívar, Barranquilla 080005, Colombia; yolanda.vega@unisimon.edu.co (Y.V.-S.); carlos.espejo@unisimon.edu.co (C.A.E.); steffen.cantillo@unisimon.edu.co (S.C.); luis.gaviria@unisimon.edu.co (L.G.); jheifer.paez@unisimon.edu.co (J.P.); carlos.ochoa@unisimon.edu.co (C.O.);; 2 Centro de Investigación e Innovación en Biodiversidad y Cambio Climático, Adaptia, Universidad Simón Bolívar, Barranquilla 080005, Colombia; sindy.chamorro@unisimon.edu.co; 3 Systems Engineering Department, Universidad del Norte, Barranquilla 081007, Colombia; 4 Centro de Investigación en Ciencias de la Vida, CICV Universidad Simón Bolívar, Barranquilla 080005, Colombia; roberto.pestana@unisimon.edu.co; 5 Solenium S.A.S., Medellin 050031, Colombia; camilom@solenium.co

**Keywords:** smart sensing, artificial intelligence, smart cities, electrical failure, SVM, failure detection

## Abstract

Electrical power systems are crucial, yet vulnerable, due to their complex and interconnected nature, necessitating effective fault detection and diagnostics to ensure stability and prevent disruptions. Advances in artificial intelligence (AI) and the Internet of Things (IoT) have transformed the ability to identify and resolve electrical system problems efficiently. Electrical systems operate at various scales, ranging from individual households to large-scale regional grids. In this study, we focus on medium-scale urban infrastructures. These environments present unique electrical challenges, such as phase imbalances and transient voltage fluctuations, which require robust fault detection mechanisms. This work investigates the use of AI with dynamic programming and a support vector machine (SVM) to improve fault detection. The data collected from voltage measurements in urban office buildings with smart meters over a period of six weeks was used to develop an AI model, demonstrating its applicability to similar urban infrastructures. This model achieved high accuracy in detecting system failures, identifying them with a performance greater than 99%, highlighting the potential of smart sensing technologies combined with AI to improve urban infrastructure management. The integration of smart sensors and advanced data analytics significantly increases the reliability and efficiency of energy systems, promoting sustainable and resilient urban environments.

## 1. Introduction

### 1.1. Overview

Electrical power systems are complex and dynamic, making them vulnerable to disturbances and malfunctions [1]. This vulnerability arises from reliance on large-scale generation plants [2] and interconnected networks, where failures can propagate rapidly [3]. Accurate fault detection and classification are critical to enabling swift corrective actions and preventing disruptions [4]. Key concerns include identifying the conditions that trigger disturbances, pinpointing vulnerable components, and understanding the role of network structures in fault propagation [2]. Failure to act promptly can lead to local outages or widespread blackouts, causing economic losses and safety risks [3].

Fault detection methods range from basic visual inspections to advanced AI-driven diagnostics. Traditional approaches, such as manual inspections, help to identify defects like damaged cables [5]. Sensor-based monitoring systems track real-time parameters [6]. However, artificial intelligence (AI) has revolutionized fault detection, minimizing human involvement while enhancing accuracy [7]. AI-powered systems analyze real-time electrical data, identifying anomalies related to voltage, current, and resistance [8]. These technologies enhance response speed, reduce downtime, and improve system reliability [9].

Latin America faces significant challenges in electrical fault management, particularly in transmission and distribution networks. Utilities struggle with non-supplied energy (NSE) penalties due to medium voltage (MV) faults [10], and deregulation has exacerbated inefficiencies in fault detection [11]. High seismic activity in regions like the Andes further complicates grid stability [12]. Countries such as Chile and Colombia experience recurring disruptions, highlighting the need for advanced monitoring solutions. Historical blackouts, such as the 2007 Colombian outage, demonstrate the risks of inadequate fault management [13]. AI-driven fault detection is transforming energy systems by enabling real-time data processing for demand forecasting, grid optimization, and predictive maintenance [9]. Deep learning models enhance decision making for energy management, integrating renewable energy sources and supporting smart grids [14]. Table 1 summarizes the advantages of AI-based fault detection over traditional fault detection, which highlights the contributions that these technologies can make in this field. AI techniques improve efficiency and reliability, playing a crucial role in the development of intelligent energy infrastructures for smart cities [15].

Electrical systems operate at various scales, from city-wide power grids to individual infrastructures, including residential, commercial, and industrial buildings. Fault detection is crucial to ensure a stable power supply, particularly in urban environments where energy demand is high. In this work, we develop a method for electrical fault detection focused on urban office buildings, which serve as a representative case of medium-scale infrastructures equipped with smart meters. These buildings experience common electrical issues such as undervoltage, overvoltage, and phase imbalance, making them a suitable testbed for AI-driven fault detection. A smart meter developed in Colombia (Quoia device) was deployed across multiple administrative office buildings with three-phase AC power, collecting several power-related variables over six weeks. The proposed method combines a pre-processing stage with dynamic programming to highlight power series fragments with anomalies and a GPU-optimized support vector machine algorithm to classify eight fault types. While the study focuses on office buildings, the methodology is applicable to similar urban structures with smart metering systems, such as commercial centers, hospitals, and educational institutions, ensuring scalability and adaptability in urban electrical grids.

### 1.2. Previous Work

Numerous studies have explored the application of artificial intelligence (AI) for predicting electrical faults. For instance, in the work by Mittal et al. [16], the authors evaluated the effectiveness of decision tree-based classifiers in detecting and classifying various types of electrical faults, such as phase-to-phase faults (e.g., BC), three-phase faults (ABC), single-phase-to-ground faults (AG), three-phase-to-ground faults (ABCG), and phase-to-phase-to-ground faults (BCG). This approach is notable for its computational simplicity and interpretability. However, its scalability may be limited when applied to more complex electrical systems or scenarios with imbalanced data.

Another study applying machine learning techniques is the one presented by Vivek et al. [17]. This research focused on enhancing the efficiency and accuracy of fault diagnosis by employing decision tree classifiers and random forest algorithms. The methodology emphasized the importance of addressing imbalanced datasets through the use of the synthetic minority over-sampling technique (SMOTE) to improve model performance. The proposed approach covered various fault types, including line-to-line faults (AB), line-to-ground faults (AG, BG), three-phase faults (ABC), three-phase-to-ground faults (ABCG), and normal operating conditions (no fault). The study was validated using diverse datasets representing these scenarios, showcasing the effectiveness of machine learning techniques in real-time fault detection and localization.

A different machine learning approach is presented by Naik and Koley [18]. This work introduced a fault detection and classification scheme for AC and HVDC transmission lines using the k-nearest neighbors (KNN) algorithm. The proposed method was applied to a hybrid AC/HVDC transmission system integrated with a doubly fed induction generator (DFIG) wind farm. The model employed discrete wavelet transform (DWT) for feature extraction and used KNN classifiers to detect and classify faults under various conditions. The study specifically addressed AC faults, including single line-to-ground (AG, BG, CG), line-to-line (AB, BC, CA), and three-phase-to-ground (ABCG), as well as HVDC faults like pole-to-ground (P-G) and pole-to-pole (P-P) faults. Extensive simulations validated the method’s performance, achieving 100% accuracy in fault detection and classification. While promising, this result should be interpreted with caution, as real-world conditions often introduce noise, equipment variability, and unforeseen scenarios that may expose limitations that are not evident in controlled simulations.

Other works have applied deep learning to the problem of fault prediction. In the study by Kumar et. al. [19], the authors proposed a hybrid model based on a support vector machine (SVM) and convolutional neural network (CNN) for fault detection in electrical transmission systems. The approach focused on quickly and accurately identifying faults through a system that combined traditional classification methods (SVM) with advanced deep learning capabilities (CNN). The model specifically addressed the classification of high impedance faults (HIF) and non-HIF using feature selection techniques and range-Doppler images generated from voltage and current data.

Another study utilizing deep learning is by Shang [20]. This work investigated a deep learning-based approach for simulating fault detection and prediction in electrical power systems. The study employed recurrent neural networks (RNNs), gated recurrent units (GRUs), and bi-directional GRUs (Bi-GRUs) enhanced with an attention mechanism to analyze time-series data, such as voltage and current fluctuations during fault scenarios. Using these techniques allowed the author to improve the processing ability of long sequence data by introducing a gating mechanism at a lower cost, which led to more efficient use of computing resources. The model was trained and tested on simulated power grid data to identify potential faults, enhancing the reliability and robustness of power systems, and it achieved 97.1% accuracy. The use of Matlab/Simulink, while being a robust solution, limited its applicability and/or deployment due to their environmental requirements. Nevertheless, its workflow served as an inspiration for us to try and implement it on more accessible platforms. Table 2 summarizes the most relevant previous work, including each model’s best performance.

Furthermore, other authors, such as Li [21], have revealed that another area of great interest is the application of AI in the real-time monitoring of complex electrical systems. These authors state that through the integration of advanced sensors and machine learning algorithms, it is possible to obtain a proactive view of the status of the equipment. This capability allows not only detecting faults in real time but also predicting their occurrence, facilitating the implementation of predictive maintenance actions. As a result, the authors point out that energy efficiency is optimized, the useful life of assets is extended, and greater system reliability is guaranteed.

The state of the art described contributes significantly to the methodology proposed in this study, providing a deep context on current developments and highlighting the impact of advanced artificial intelligence (AI) techniques especially in the detection of electrical faults. The papers described share a common goal: detecting electrical failures using machine learning or deep learning techniques. While our objective aligns with theirs, we incorporate an additional methodology. We preprocess the voltage signal to identify atypical values, then apply machine learning classification to these segments. This approach reduces computational costs, making the process more practical for real-world applications. The integration of smart meters, sensors, and connected devices enables the collection of comprehensive real-time data, creating an extensive information network that allows AI to fully leverage its capabilities to predict and identify potential failures within energy systems for smart sensing and smart city applications.

Most prior studies fail to incorporate strategies aimed at reducing computational costs, limiting the efficiency and real-world applicability of their solutions. In our paper, we employ dynamic programming (using the PELT algorithm) to filter data segments exhibiting abnormal behavior. This preprocessing step is followed by classification using a fast support vector machine (SVM) model. The filtering stage significantly reduces the system’s processing load, enhancing its practicality for real-world applications. While authors like Shang [20] have explored similar issues, their approaches have not been applied to real-world data. Our study not only introduces a novel strategy but also demonstrates its effectiveness in a real-world context. Furthermore, our results outperform existing state-of-the-art methods using non-simulated data. We also address a broader range of electrical failures—such as undervoltage, overvoltage, and voltage imbalance—that are often overlooked in previous studies.

In this context, the present study used dynamic programming and a support vector machine (SVM) to develop an AI model designed to identify possible failures in electrical systems. An electronic device named Quoia, which measures current and voltage, gathered 5 million data points from office buildings over one and a half months. This dataset was used to train the model, which achieved high accuracy, showing the high potential of this model and methodology to be implemented for smart sensing in electronic devices.

## 2. Materials and Methods

In this study, we apply a new methodology that combines dynamic programming with a support vector machine to predict electrical faults in power data. Our process involves data collection, data pre-processing, and training and testing of two models.

### 2.1. Data Collection

For the data collection, a Quoia [22] device was used, which was developed by the Colombian company Solenium S.A.S in Medellin, Colombia. The dataset utilized in this study is not publicly available due to proprietary restrictions. However, it can be accessed upon reasonable request by contacting the corresponding author or directly to the company Solenium S.A.S. through their official website https://solenium.co/.

**Figure 1:**
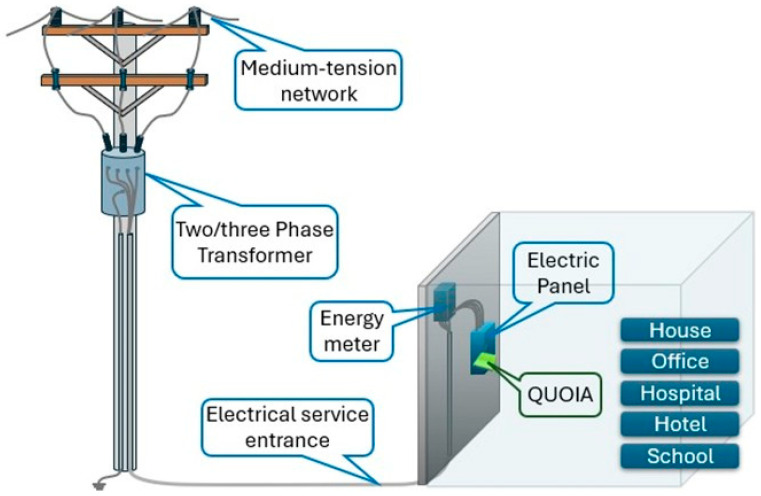
Scheme representation of how the Quoia device is connected.

The device was connected to a three-phase AC circuit and it was capable of capturing each phase current and voltage. From this data, we calculated active power or real power and reactive power. For this study, the device was installed between the main distribution power supply and the building to inspect (house, hotel, hospital, etc.), as shown in Figure 1.

The model was trained with the dataset spanning from 21 September 2021 to 20 October 2022, with data collected at one-minute intervals throughout the specified period. For this study, only office buildings were considered, specifically 10 office buildings located in Medellin, Colombia. The obtained data were from a real scenario. The Quoia device was installed in 10 office buildings and 5 power variables for three phases were measured for 13 months and stored in a cloud server. Then these observations were filtered and downloaded for analysis. The power failures detected in the dataset corresponded to real failures in the three-phase power system.

The original dataset had 5,398,640 observations from 15 variables, organized into 5 main variable groups: vp (phase voltage), cp (phase current), app (active power), rpp (reactive power), and pfp (power factor). Each group included three variables corresponding to the three electrical phases, resulting in a total of 15 variables.

The 5 main variables of each group were related to each other in each of the phases, having active power and reactive power variables that depended directly on the product of voltage and current and the power factor variable, which in turn depended on the deviations of the phase angles of the voltage and current signals. Due to the above and with the purpose of reducing the number of variables and the computational load of the system, only the three phase voltages were considered as main variables for this study, since they allowed the identification of electrical faults in the supply of the three-phase system and, in turn, the faults associated with the currents and/or loads ended up affecting the voltage of the respective phase. In the final detection model, only three variables were considered: the voltage (vp) of each of the three phases was included for training the model. To determine whether there was a power failure, for this study and according to the power quality parameters established in the Colombian NTC5001 Standard of 2008 [23] and Resolution 065 of 2012 of the Energy And Gas Regulation Commission, an electrical anomaly or failure was considered when the input voltage exceeded variations of +/−10% of the nominal voltage (120 V) for one minute, which in our case corresponded to voltages higher than 132 V RMS and lower than 108 V RMS. The standard additionally established the type of anomalies, such as undervoltage, overvoltage, voltage imbalance, etc.

**Figure 2:**
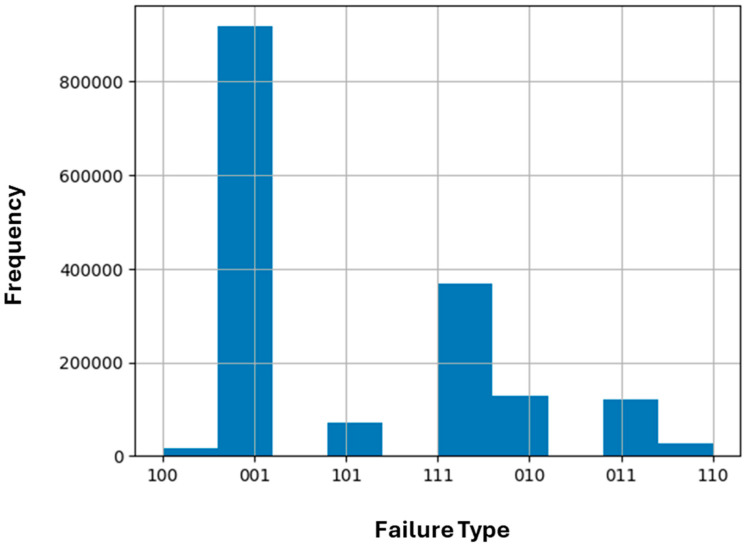
Histogram of failure types in the complete dataset without normal cases.

Failures were labeled with a three digit code. Each digit represented the voltage in one phase. If the digit was 1, it meant that there was a failure in that phase, and if it was 0, then the voltage was within acceptable limits. So, for example, an observation labeled 010 meant there was a failure in the second phase. The distribution of failures for the dataset is presented in Table 3 and as a histogram excluding normal cases in Figure 2.

With this dataset, we performed a multiclass classification with one target variable: one of the eight failure type classes. As can be seen in Table 3, this was an imbalanced dataset since most of the observations were of normal voltage in the three phases. The most frequent type of failure was 001, which occurred when a failure was present in the third phase voltage. As will be discussed in the following sections, we obtained very good results without the need to apply special techniques to handle the imbalance; however, this is one of our goals for future work.

### 2.2. Quoia Device

The Quoia device [22] is an energy meter designed by Solenium to comply with the regulations and standards governing the monitoring and control of electrical energy at generation and commercial boundaries. It is compatible with single-phase, two-phase, and three-phase installations, measuring both active and reactive energy, with a nominal operating range of 60 Hz. It features non-volatile memory storage and performs automatic data reading and processing.

Additionally, it is equipped with voltage and current input filters, with a cut-off frequency of −3 dB at 72.02 kHz. The system allows for the identification of energy consumption and real-time visualization of active power, with update intervals ranging from one to ten seconds. The device is integrated with a cloud platform and is installed across various sectors, including textiles, brick manufacturing, healthcare, residential, food services, social clubs, infrastructure product manufacturing, schools, offices, universities, and solar energy.

### 2.3. Model Development

Optimization problems arise in numerous scientific and engineering domains, necessitating the development of efficient algorithms for their resolution. Dynamic programming (DP) is a fundamental technique that decomposes complex problems into smaller overlapping subproblems, solving each subproblem once and storing its solution to avoid redundant computations. This method is widely applied across various disciplines, including electrical engineering, operations research, and artificial intelligence, to optimize decision-making processes and enhance computational efficiency.

Dynamic programming (DP) was formalized by Richard Bellman in the 1950s [24], stating that an optimal solution to a problem can be constructed from optimal solutions to its subproblems. This recursive structure enables dynamic programming algorithms to solve problems by breaking them into manageable stages, used commonly in, for example, route optimization in power grids to minimize transmission losses, scheduling of energy generation to balance supply and demand efficiently, or shortest path problems in network routing and logistics. Classical DP methods utilize recursion with memorization or tabulation. For example, in power system optimization, DP is used to minimize the cost of power generation while meeting demand constraints. The unit commitment problem, which determines the optimal schedule for power generators, has been efficiently solved using DP-based approaches [25]. In this work, a dynamic programming approach, the pruned exact linear time (PELT) algorithm was used as a change point detection method. Change point detection is the task of finding changes in the underlying model of a signal or time series. Detection methods are expressed as the combination of the following three elements [26]:

- Cost function: The cost function is a measure of homogeneity, and it is expected to be low if the sub-signal is “homogeneous” (it does not contain any change point) and large if the sub-signal is “heterogeneous” (it contains one or several change points).
- Search method: The search method is the resolution procedure for the discrete optimization problems associated with the problem of finding a known or unknown number of changes in a signal.
- Constraint (on the number of change points): When the exact number of changes is unknown, a constraint is introduced in the form of a complexity penalty. The selection of this penalty is closely related to the magnitude of the changes to be detected. If the penalty is too low, numerous change points may be identified, including those caused by noise. On the other hand, an excessively high penalty may result in detecting only the most significant changes—or even none.

**Figure 3:**
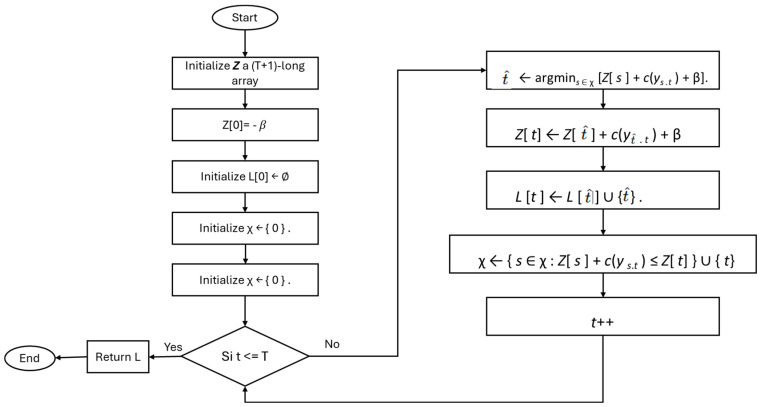
Flow diagram of PELT algorithm.

The PELT algorithm defines a new search method when the number of change points is unknown and the penalty function is linear. This approach considers each sample sequentially and, thanks to an explicit pruning rule, may or may not discard it from the set of potential change points. Given a signal { y t }T t = 1, cost function c(·), and penalty value β. The process followed by the PELT algorithm is explained in Figure 3 [26].

**Figure 4:**
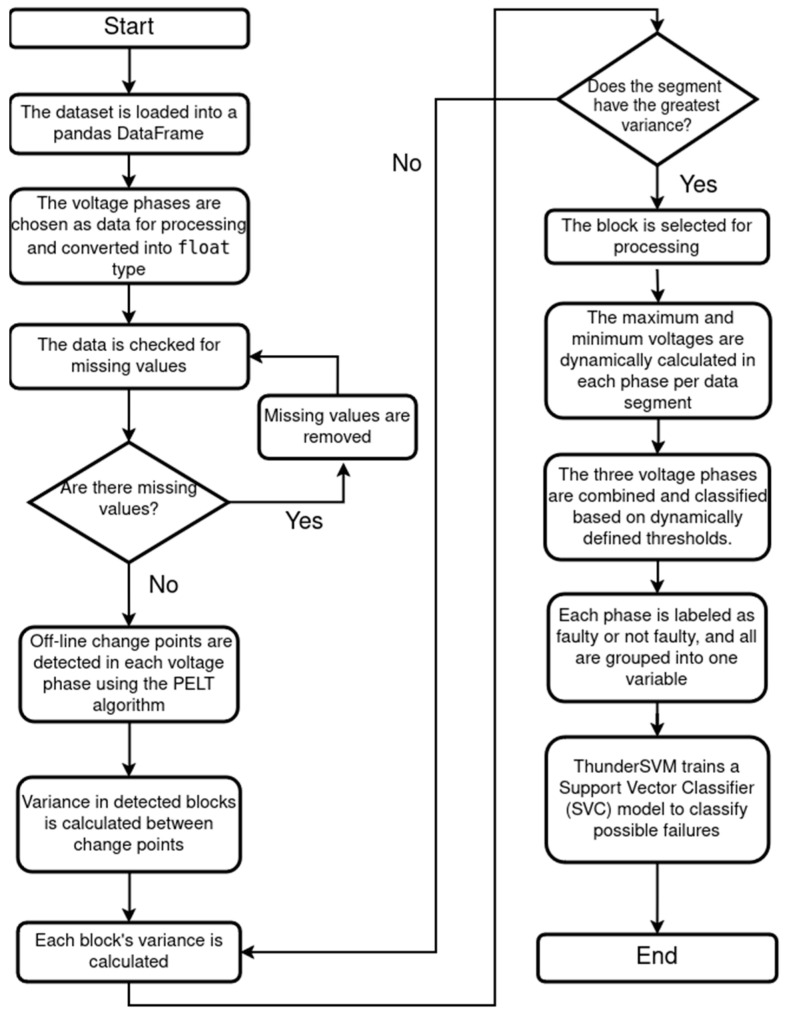
Schematic representation of how the data were curated and processed to develop the model. In the initial stage, the data are loaded into a Pandas DataFrame, where voltage phase data are converted into a float data type. Then the algorithm implements a systematic check for missing values, removing data across all phases simultaneously to maintain consistent temporal alignment. For the analysis stage, the voltage phase data are processed using the PELT algorithm to detect off-line changes, which divide the data into blocks. Then the algorithm calculates and evaluates the variance of each block, selecting those with the highest variance. Within the selected blocks, the maximum and minimum voltages are dynamically calculated for each phase. Finally, each phase is labeled as “faulty” or “not faulty” and grouped into a single variable, which is used to train the support vector classifier (SVC) model.

This study combined dynamic programming with an SVM to generate an AI model to identify potential failures within energy systems. The schematic representation of how the data were curated and processed for the model is presented in Figure 4.

The model was developed using Python language version 3.10. Initially, all of the data were loaded using an csv file and then structured into a Pandas DataFrame for easy manipulation and cleaning. Voltage data across different phases were converted to float types to ensure consistent numerical formatting. The dataset was checked for any missing values, which were removed to maintain data integrity.

In the first stage, to detect and analyze non-stationary segments in the data, the Pelt algorithm was used to identify off-line change points within each voltage phase. The variance in each detected segment was calculated to locate the segment with the greatest variation, indicating potential abnormalities. Next, the maximum and minimum voltage values were dynamically calculated per segment to establish thresholds for classifying each phase as either normal or faulty. Using these thresholds, all three voltage phases were combined and labeled accordingly to indicate fault presence. Then, in the second stage, this labeled dataset was then used to train a support vector classifier (SVC) model with ThunderSVM, leveraging GPU acceleration for efficient processing. The resulting model effectively classified potential failures, providing a robust tool for fault detection based on voltage fluctuations across phases.

For the first stage of the process, the data were analyzed using the ruptures Python library [26], which can be used for off-line change point detection. This library provides a collection of methods for the analysis and segmentation of non-stationary signals. This library creates an instance in an implementation of pruned exact linear time (PELT). The PELT algorithm is a DP-based technique for change point detection, which is instrumental in detecting changes in time-series data and widely applied in fault detection and anomaly identification. It is commonly used for identifying voltage fluctuations in electrical networks, detecting faults and disturbances in power grids, and monitoring and diagnosing equipment failures. This algorithm computes the segmentation of the data in a way that minimizes the constrained sum of approximation errors [27] and can detect changes based on the properties of the data from a statistical perspective. Let us assume *y_p_*_=1:*h*_ = (*y*_1_, *y*_2_,*y_h_*) is the ordered sequence data with *h* number of data points. These data contain *m* number of change points at positions *τ_q_*_=1:*m*_ = (*τ*_1_, *τ*_2_, …,*τ_m_*) and these *m* change points split the data into *m* + 1 segments. The *qth* segment data are given as $y_{\left(\tau_{\left(q-1\right)}+1:\tau_{q}\right)}$. A common approach of this method is based on minimizing the cost-based Equation (1) [28]:

(1) $$\sum_{q=1}^{m+1}\left[C\left(y_{\left(\tau_{\left(q-1\right)}+1:\tau_{q}\right)}\right)+\beta \right]$$

where *y* is the time series, *C* is the cost function, β is the penalty against over fitting, *τ* is the vector of change positions, and *i* is the *i*th point in the time series. The PELT method takes Equation (1) and improves the computational efficiency using a pruning process, giving an optimal segmentation *F*(*n*):

(2) $$F\left(n\right)=\begin{array}{c} \min\\ \tau_{m} \end{array}\left\{F\left(\tau_{m}\right)+C\left(y_{\left(\tau_{m}+1:h\right)}\right)+\beta \right\}$$

The settings for the PELT model were the model as *l*2 and min_size as 500,000, through the ruptures 1.1.9 Python library. The results of the changes were used as input to train the model using the ThunderSVM Python library. ThunderSVM is an efficient and open source SVM software toolkit that exploits the high-performance of graphics processing units (GPUs) and multi-core CPUs. It supports all of the functionalities—including classification (SVC), regression (SVR), and one-class SVMs—and uses identical command line options. Previous experimental results showed that ThunderSVM is generally an order of magnitude faster than LibSVM while producing an identical SVM [29]. In order to train the model with ThunderSVM, the resulting dataset was split into fixed train and test datasets, where 80% of the observations were selected for the training dataset and the remaining 20% were included in the test dataset. The observations were selected randomly from the 11 offices. After this initial test dataset, the model was also tested with the whole data of a single unseen office. The ThunderSVM library brings a solution to the need for computer power to performs SVM models on large and complex systems. ThunderSVM uses both GPUs and CPUs for training the SVM model, giving an extra boost, thereby improving the training time. The settings for the ThunderSVM model were C to 100, which helped to avoid overfitting in the model, and for the kernel rbf (radial basis function) was set.

### 2.4. Evaluation Metrics

Several metrics were used to evaluate the performance of the predictions in the three stages. Regarding the metrics applied, it is worth highlighting that the confusion matrix was presented as an essential tool that summarizes the distribution of observations into four defined categories. Most performance metrics in classification problems are derived from the confusion matrix. This is a matrix in which each row represents the instances in the actual class and each column represents the predictions of each class [30].

**Figure 5:**
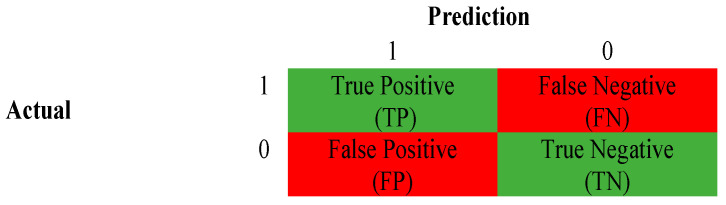
Confusion Matrix.

As can be seen in Figure 5, observations can be classified into one of four categories:

- True positive (TP): The model predicted the positive class and the observation was truly positive.
- True negative (TN): The model predicted the negative class and the observation was truly negative.
- False positive (FP): The model predicted the positive class, but the observation was actually negative. This means that the observation was classified as positive incorrectly.
- False negative (FN): The model predicted the negative class, but the observation was actually positive. In this case, the observation was erroneously classified as negative [30].

From these four categories, it is possible to calculate various evaluation metrics, with accuracy being the most used. The accuracy (ACC), which is calculated using Equation (3), provides an overview of what percentage of observations were correctly classified, both in negative and positive cases. Its mathematical expression is formulated as follows:

*ACC* = (*TP* + *TN*)/(*P* + *N*) = (*TP*+*TN*)/(*TP* + *FP* + *TN* + *FN*) (3)

The recall, sensitivity, or true positive rate metric, shown in Equation (4), is a metric that evaluates the model’s ability to accurately detect positive cases. Its mathematical expression is defined as follows:

*Recall* = *TP*/*P* = *TP*/(*TP* + *FN*) (4)

Another metric is specificity, defined in Equation (5), also known as the true negative rate (TNR), which is a metric used to evaluate the model’s ability to accurately identify negative cases. This metric is key in evaluating model performance in situations where accuracy in detecting negative cases is of high importance. Its mathematical expression is defined as follows:

*Specificity* = *TN*/*N* = *TN*/(*TN* + *FP*) (5)

On the other hand, precision, defined in Equation (6), also known as the positive predicted value (PPV), constitutes a fundamental metric in evaluating the model’s ability to determine precisely how many of the cases classified as positive are, in fact, truly positive.

*Precision* = *TP*/(*TP* + *FP*) (6)

Finally, the F1 score in Equation (7) is a fundamental metric, representing the harmonic mean of precision and sensitivity. Its calculation is based on the harmonization of these two key metrics, thus offering a comprehensive evaluation of the model’s performance:

F1 Score = 2 × (PPV × TPR)/(PPV + TPR) = 2TP/(2TP + FP + FN)(7)

## 3. Results

**Figure 6:**
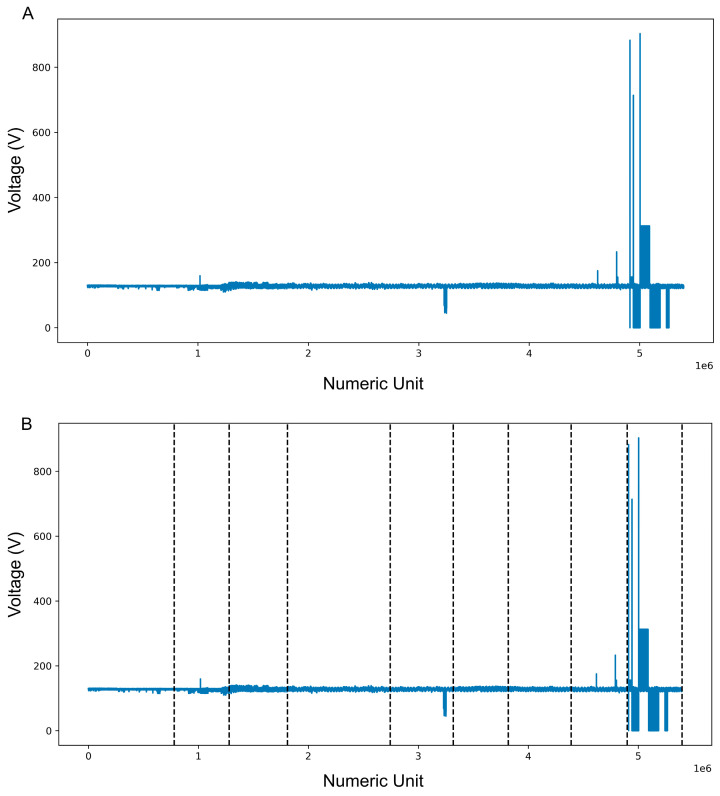
Plot of the voltage data used to build the model. (**A**) Voltage data plotted as captured by the device. (**B**) Voltage data with the divisions identified by the ruptures library.

As can be seen in Figure 6, the data were used as input for rupture and the black dotted lines represent where this library considered a change in the signal. Numeric unit refers to the ID or sequence number within the captured voltage phase.

These changes recognized by rupture were used to train the model using the SVM scheme through ThunderSVM, and the classification report from this model is presented in Table 4. In this table, each row refers to one class used by the model, where each number refers to a phase; 0 and 1 refer to non-failure and failure, respectively, so the first row 000 indicates non-failure in the three phases. Precision quantifies the percentage of accurate classifications for a given class in relation to all classifications made for that class. Recall, also referred to as sensitivity, quantifies how many actual instances of a class the model correctly identified. The F1 score, which is the harmonic mean of precision and recall, offers a balanced metric that evaluates both the precision and the sensitivity of the model. These metrics provide a holistic view of the model’s performance.

These metrics offer a detailed evaluation of the model’s ability to detect and classify electrical faults accurately. It is crucial for gauging the model’s performance in aligning identification fault classes with their actual counterparts. The report distinctly illustrates the model’s precision for each class (different combinations of failures and non-failures in each phase) and its effectiveness in reducing the number of false positives. High precision across the dataset underscores the model’s operational efficacy under controlled conditions. Additionally, a high recall value in the matrix is vital, as it shows the model’s capability to capture all factual instances of faults, a critical factor in applications where reliability is paramount. The F1 score, by integrating precision and recall, provides a cohesive metric that effectively balances accurate fault detection with precision. Furthermore, the ’support’ metric in the matrix, indicating the number of instances for each class within the dataset, offers insights into the data volume the model was tested and validated on, demonstrating its adaptability to a range of fault scenarios.

The analysis indicated that the model performed well across most classes, achieving high marks in precision, recall, and F1 score. However, performance dropped significantly for class 101, where the precision was 0.72, meaning that 72% of the instances the model identified as class 101 were correctly classified. By contrast, the recall for class 101 was only 0.08, suggesting that the model identified only 8% of all genuine class 101 instances. This pointed to difficulties in accurately detecting class 101 within the dataset used.

To prove the model, a new dataset was used. A dataset from a different office building was utilized, this time with 362,518 data of voltage. The classification report is presented in Table 5. The findings presented highlight the model’s high performance across all classes, with perfect scores in precision, recall, and F1 score for most classes. However, a small performance drop was observed for classes 010 and 111, but they still had a precision and F1 score above 0.95. Despite this, the overall accuracy of the model remained high, demonstrating its ability to make correct identifications in most cases.

In this second stage, the model was evaluated using data not seen during training, offering crucial insights into its generalization capability and performance under varied conditions. This analysis confirmed the model’s consistency, with a high precision and F1 score across multiple classes, highlighting its ability to maintain robust performance even when faced with new data. Such consistency proved the model’s practical utility in real-world applications, where conditions and types of faults can vary significantly. The consistency in high recall and F1-score values across different datasets indicated that the model not only learned effectively but also applied its learning to make accurate and reliable identifications in diverse scenarios.

This detailed analysis of each classification report underscored the model’s technical competence and validated its practical applicability in electrical systems, where quick detection, classification, and response to faults are essential for operational stability and safety. Thus, the model’s ability to operate accurately in various situations reinforced its value as a reliable tool in the detection and classification of electrical faults.

## 4. Discussion

The proposed methodology for fault detection was evaluated in two stages: first with a test dataset from the same nodes as the training data, and then in a second stage, with completely new data from a different office building. In the first stage, an accuracy of 0.99 was achieved, which demonstrated almost perfect classification. The 0.01 that was misclassified was probably due to the very small number of observations with that class in the training data, which did not allow the model to make a good generalization. For future work, synthetic data may be used to train the model in less frequent fault types.

In the second stage, again an accuracy of 0.99 was achieved, which demonstrated that our model can be applied to data from different sources and still achieve high performance. Again, the classification was not perfect due to the fault types that had relatively few cases in the training dataset. This demonstrated the importance of having more data to train a more robust model. On the other hand, synthetic data may be a viable option for future research.

As can be seen in Table 6, if we compare our results to others from the state of the art, our model outperforms most of the other models, except for the model presented by Naik and Koley [18]. This model achieves perfect accuracy; however, this work is tested on simulated data, while our model is evaluated in a real-world scenario. If we compare our performance with others on real data, our model has higher accuracy.

The integration of these models into a smart device such as Quoia, or its associated web platform, will lead to real-time detection of the most frequent electric fault types, which will help to prevent serious economic losses and help to create more efficient and safe buildings. In this case, for practical deployment, it is recommended that smart meters, typically built with microcontrollers featuring IoT capabilities, be prioritized for fast and delay-free acquisition of electrical signal measurements. These devices should leverage their wireless capabilities to transmit the metering data to a high-performance server over a Wi-Fi connection using the TCP/IP protocol, where the proposed fault detection model is executed.

## 5. Conclusions

In this paper, a method for detecting electrical faults that combines dynamic programming and support vector machines is proposed and assessed. The findings from this research based on the model’s results support the effectiveness of the methodology in detecting and classifying electrical faults, highlighting its significance in enhancing the reliability and safety of electrical power systems.

The proposed model demonstrated a high accuracy rate, achieving 99% accuracy in fault detection within three-phase systems. The AI-based approach effectively classified electrical anomalies such as overvoltage, undervoltage, and phase loss, ensuring a more reliable fault diagnosis process compared to traditional methods.

Therefore, these findings lay a solid foundation for future research in this field aimed at further improving the quality and efficiency of the electrical supply for the benefit of communities and the industry.

The study conducted to detect and classify electrical faults using dynamic programming and a support vector machine (SVM) yielded encouraging results, underscoring the efficacy and applicability of this methodology in electrical power systems. The application of pattern recognition techniques, such as SVM, enabled the differentiation between faulty and healthy electrical systems, even pinpointing the specific phase affected in three-phase systems. This method proved to be efficient in detecting, classifying, and locating faults within the study. The model achieved a precision of 99% when measuring the presence of fault within any of the three phase voltages, in this specific case, demonstrating its capability to accurately classify electrical faults. These outcomes exceeded initial expectations, confirming the utility and potential of artificial intelligence, particularly in the detection and classification of electrical faults in power systems. Future research perspectives include exploring the adaptability and performance of the model across different datasets, as well as its effectiveness in more complex classification challenges within electrical engineering. A comparison with existing state-of-the-art models revealed that the proposed approach surpasses decision tree-based methods (88.95% accuracy) and random forest classifiers (83.49%), demonstrating its robustness in real-world conditions. While some simulated models achieved 100% accuracy, their dependency on synthetic datasets limits their practical deployment, reinforcing the applicability of our method in real electrical networks. Additionally, it is suggested to perform comprehensive comparisons with other machine learning methods combined with dynamic programming and an advanced SVM to assess their performance across a broader spectrum of applications. Another issue to consider for future work would be to include other variables in the model, such as current, ambient temperature, and phase cable temperature measurements. These enhancements would help to strengthen the system by enabling the identification of issues like overheating due to faulty connections.

## Figures and Tables

**Table 1:** Comparison between traditional and AI-based fault detection.

Feature	Traditional Fault Detection	AI-Based Fault Detection
Methodology	Manual inspections, visual checks, sensor-based monitoring	AI-driven data analysis, real-time monitoring, deep learning models
Accuracy	Limited by human error and sensor capabilities	High accuracy due to AI-powered anomaly detection
Speed of Detection	Slower, depends on scheduled inspections or sensor alerts	Real-time fault prediction and instant anomaly detection
Human Involvement	High, requires expert analysis and intervention	Minimal, automated fault classification and response
Predictive Capabilities	Low, mostly reactive to faults after they occur	High, capable of predicting potential failures in advance
Scalability	Limited to available workforce and sensor coverage	Scalable across large and complex power grids
Integration with Smart Grids	Limited, relies on standalone monitoring tools	Seamless integration with smart grids and automation systems

**Table 2:** Fault prediction previous work summary.

Authors	Techniques	Data Source	Performance
Mittal et al. [16]	Decision Tree Classifier	Simulated data	88% accuracy
Kumar et al. [19]	Hybrid SVM-CNN Model	Real-world and simulated data	Hybrid Model: 95.70% accuracy
Vivek et al. [17]	Decision Tree, Random Forest, SMOTE	Real-world dataset with SMOTE augmentation	Decision Tree: 88.95%, Random Forest: 83.49%
Shang [20]	Bi-GRU with Attention	Simulated data (MATLAB/Simulink)	97.1% accuracy
Naik and Koley [18]	KNN, DWT	Simulated data (MATLAB/Simulink)	100% accuracy

**Table 3:** Failure type distribution in complete office dataset.

CLASS	COUNT
000	3,750,930
001	917,815
010	129,225
011	120,419
100	15,931
110	25,509
101	70,414
111	368,397
TOTAL	5,398,640

**Table 4:** Results on the initial test dataset.

Test	Accuracy	Recall	F1 Score	Support
000	1.00	1.00	1.00	126,008
001	0.97	1.00	0.98	40,016
010	0.99	1.00	0.99	10,332
011	1.00	0.99	0.99	4711
100	0.97	0.99	0.98	430
101	0.72	0.08	0.14	1701
110	0.99	0.99	0.99	1776
111	0.98	1.00	0.99	16,910
010	0.99	1.00	0.99	10,332
Accuracy			0.99	201,884
Macro avg	0.95	0.88	0.88	201,884
Weighted avg	0.99	0.99	0.99	201,884

**Table 5:** Results of the trained model on different office data.

Class	Precision	Recall	F1 Score	Support
000	1.00	1.00	1.00	325,995
001	1.00	1.00	1.00	68
010	0.97	1.00	0.99	28,640
011	1.00	1.00	1.00	5256
100	1.00	1.00	1.00	4
110	1.00	1.00	1.00	2008
111	0.96	0.99	0.97	547
accuracy			1.00	362,518
macro avg	0.99	1.00	0.99	362,518
weighted avg	1.00	1.00	1.00	362,518

**Table 6:** Comparison of our method with previous models.

Authors	Data Source	Performance
Mittal et al. [16]	Simulated data	88% accuracy
Kumar et al. [19]	Real-world and simulated data	Hybrid Model: 95.70% accuracy
Vivek et al. [17]	Real-world dataset with SMOTE augmentation	Decision Tree: 88.95% Random Forest: 83.49%
Shang [20]	Simulated data (MATLAB/Simulink)	97.1% accuracy
Naik and Koley [18]	Simulated data (MATLAB/Simulink)	100% accuracy
Our model	Real-world dataset	99% accuracy

## Data Availability

Data is contained within the article.
